# Evaluation of Bioinformatic Programmes for the Analysis of Variants within Splice Site Consensus Regions

**DOI:** 10.1155/2016/5614058

**Published:** 2016-05-24

**Authors:** Rongying Tang, Debra O. Prosser, Donald R. Love

**Affiliations:** Diagnostic Genetics, LabPLUS, Auckland City Hospital, P.O. Box 110031, Auckland 1148, New Zealand

## Abstract

The increasing diagnostic use of gene sequencing has led to an expanding dataset of novel variants that lie within consensus splice junctions. The challenge for diagnostic laboratories is the evaluation of these variants in order to determine if they affect splicing or are merely benign. A common evaluation strategy is to use* in silico* analysis, and it is here that a number of programmes are available online; however, currently, there are no consensus guidelines on the selection of programmes or protocols to interpret the prediction results. Using a collection of 222 pathogenic mutations and 50 benign polymorphisms, we evaluated the sensitivity and specificity of four* in silico* programmes in predicting the effect of each variant on splicing. The programmes comprised Human Splice Finder (HSF), Max Entropy Scan (MES), NNSplice, and ASSP. The MES and ASSP programmes gave the highest performance based on Receiver Operator Curve analysis, with an optimal cut-off of score reduction of 10%. The study also showed that the sensitivity of prediction is affected by the level of conservation of individual positions, with* in silico* predictions for variants at positions −4 and +7 within consensus splice sites being largely uninformative.

## 1. Background

RNA splicing is an evolutionary conserved process in eukaryotic organisms for the removal of noncoding intronic regions from precursor mRNAs in order to generate mature RNAs for protein translation [1]. The differential joining of exons by alternative splicing contributes to the generation of transcript diversity [2]. Therefore, splicing is an essential step in eukaryotic gene expression and plays an important role in development and disease [3]. Splicing is performed by a class of large protein-RNA complexes called spliceosomes [1, 4]. Splicing occurs upon recognition of splice sites by spliceosomes, which catalyze the removal of the sequence/protein complex. The process of splicing is regulated by proteins comprising the spliceosome, splicing factors, and sequence elements that include the core splice signals and additional splicing enhancers/silencer sequences [4, 5].

The disruption of splicing can be caused by mutations that affect* cis*-acting regulatory elements in genes or* trans*-acting factors.* cis*-acting mutations include those that disrupt the constitutive splice sites within the gene as well as* cis*-acting elements that regulate splicing. Aberrant splicing resulting from mutation in the constitutive splice site has been described in patients with hereditary diffuse gastric cancer [6]. Mutations that disrupt* cis* splicing regulatory elements have been studied in patients with neurofibromatosis type 1 (NF1) [7].* trans*-acting mutations include those that affect the splicing machinery or factors that regulate alternative splicing. For example, rare variants in the small nuclear RNA gene* RNU4ATAC*, a gene that is essential for minor intron splicing, were identified in patients with microcephalic osteodysplastic primordial dwarfism type 1 (MOPD1) and Roifman syndrome [8, 9].

The number of known splice site mutations has expanded with the increasing availability of genetic testing. Using a combination of cDNA-PCR, denaturing high-performance liquid chromatography and multiplex ligation-dependent probe amplification, Valero et al. showed that in a cohort of NF1 patients, 22% harbor mutations with splicing defects [10]. However, the functional analysis of newly discovered variants that may affect splicing is not always feasible in a diagnostic laboratory due to the need to perform reverse transcription PCR (RT-PCR) to screen targeted transcripts. Additionally, the difficulty of obtaining tissue of interest as a source of RNA renders detection of tissue-specific transcripts challenging. Given the technical hurdles, the availability of a large dataset of splice site sequences has allowed the development of bioinformatic programmes to predict splice sites [11]. Therefore,* in silico* analysis of variants that lie within splice sites has become a commonly adopted approach to predict their potential pathogenicity.

Among the splice site prediction programmes, the most commonly used are those based on position weight matrices, which are calculated using a collection of splice site sequences and assigning a weight to each nucleotide within the sequence based on its frequency and its relative importance in the sequence motif [12]. A fundamental assumption associated with the position weight matrix-based method is position independence. In contrast, other programmes, such as the Max Entropy Scan (MES), take into account the dependencies between positions within the sequences being analysed [13]. Finally, neural network modeling programmes, such as NNSplice, NetGene2, and NANN, use a dataset of sequences to train programmes to identify splice sites [14–16].

A few studies have reported the evaluation of splice site prediction programmes, but there is no consensus guideline on the programmes that should be used and the interpretation of the prediction results. It has been suggested that the choice of programmes depends on the region of analysis. For example, it has been suggested that those programmes using the position weight matrix may be better suited to evaluate variants that activate cryptic splice sites as opposed to those lying at constitutive splice sites [17].

Evaluation by Houdayer et al. examined splice site prediction programmes using a collection of* BRCA1* and* BRCA2* gene mutations and recommended the combined use of MES and Splice Site Finder (SSF) for variant analysis [18]. However, SSF is no longer freely available, which limits its use in the routine diagnostic setting. In addition, although several studies have used MES as part of the Alamut Software Suite (Interactive Biosoftware, Rouen, France) available at http://www.interactive-biosoftware.com/doc/alamut-visual/2.6/splicing.html, Hellen has reported a significant difference in sensitivity and specificity when accessing MES via the Alamut interface compared to the free stand-alone source [19]. Additionally, a consensus cut-off value for the interpretation of a splice site score has not been determined, although a reduction of 10% in the prediction score caused by the variant has been reported in previous work [17, 19].

A study that evaluates the splice prediction programmes using the largest dataset to date was published by Jian et al. (2014) [20]. Eight splice prediction programmes were evaluated using a total of 2959 single-nucleotide variants. The programmes that showed the best performance were position weight matrix and MES accessed via the Alamut Suite. The group also developed two ensemble learning algorithms; data of their prediction has been incorporated into the dbNSFP database [21].

The aim of the study described here was to further evaluate splice site prediction programmes regarding their sensitivity and specificity in identifying disease-causing mutations and to confirm variants to be benign, which lie in splice site consensus regions adjacent to exon:intron boundaries. These regions are frequent targets for gene sequence testing in a diagnostic setting. This study focused on the major U2-type GT-AG splice sites, which constitute 98% of all splice sites [22]. The programmes selected for this study included HSF, MES, NNSplice, and Alternative Splice Site Predictor (ASSP; [23]). HSF, MES, and NNSplice were chosen based on previously published work that showed that these programmes gave better performance than others evaluated [17–19]. ASSP uses a combination of preprocessing models and backpropagation networks, using a position weight matrix for the acceptor site and a maximum dependence decomposition model for the donor site [23]. Despite its availability and ease of use, ASSP has not been evaluated in previous studies.

This study also examined the sensitivity and specificity of the prediction programmes at individual positions within the consensus splice site regions. It was shown previously that prediction for positions +3 and +5 of the donor site was more sensitive, but the remaining positions within the consensus regions were not investigated in detail [17]. The hypothesis of this study is that reliability of prediction correlates with the level of conservation at the position. The invariant dinucleotide positions GT and AG were excluded as mutations affecting these positions have been evaluated elsewhere and prediction accuracy is close to 100% due to strong level of conservation at the invariant positions [18, 19, 24]. Hellen has shown that the analyses of variants that lie further than −10 and +7 from splice junctions lose sensitivity. Therefore, the region of analysis in this study was confined to the consensus splice sequences that encompass the exon flanking positions of −9 to −3 and +3 to +7. For the purposes of our evaluation, we used a collection of 223 disease-causing mutations from Human Gene Mutation Database (HGMD® Professional) (http://www.biobase-international.com/product/hgmd) from BIOBASE Corporation and 50 benign polymorphisms from ClinVar and 1000 Genome databases [25, 26]. The result of this study aims to provide guidelines for analysis of splice variants within the consensus region in a diagnostic setting.

## 2. Materials and Methods

### 2.1. Sequence Variants

All the variants analysed in this study lay in the consensus splice region of the major U2-type introns. A total of 222 disease-causing splicing mutations (classified as “DM”) were obtained from the HGMD Professional database [27]. The mutations were divided into groups based on their positions, ranging from −9 to −3 and from +3 to +7, with each group consisting of 20 mutations (except for positions −9, −8, and +7, where only a total of 12, 18, and 13 mutations were listed in the database, resp.). This study focused on mutations that only affect the wild type (WT) splice site without activating a cryptic site. A collection of 50 polymorphisms were obtained from ClinVar and 1000 Genomes [25, 26]. The variants were selected based on the variant meeting at least one of the two criteria (1) listed in ClinVar as “benign” and/or (2) listed in 1000 Genome with a global minor allele frequency of greater than or equal to 5%. All mutations and polymorphisms used in this study are listed in Supplementary Tables available online at http://dx.doi.org/10.1155/2016/5614058.

Reference genomic sequences used for analysis were extracted from the UCSC genome browser database (Genome assembly GRCh37), which comprised the relevant exonic sequence and 100 bp of flanking intronic sequences [28].

### 2.2. Programmes and Analysis

The programmes selected for this study comprised HSF (version 2.4.1), MES, NNSplice (version 0.9), and ASSP, all of which are freely available online. Analyses using HSF and MES were performed simultaneously on the HSF website (http://www.umd.be/HSF3/), while NNSplice (http://www.fruitfly.org/seq_tools/splice.html) and ASSP (http://wangcomputing.com/assp/) were accessed individually.

The initial analyses were performed using default settings. In the event that wild type splice sites could not be predicted, the analyses were repeated with the threshold value reduced to 0 to allow for the detection of splice sites that may have lower scores than the default threshold values. The predicted scores for the reference sequences as well as the mutated sequences were recorded and the % score changes between the reference sequences and the mutated sequences were calculated according to the formula

(1) %  score  change=WT  sequence  score−mutated  sequence  scoreWT  sequence  score∗100.

Sensitivity was calculated as the proportion of reported disease-causing mutations that were predicted to be disease-causing, and specificity was calculated as the proportion of reported polymorphisms that were predicted to have no effect on splicing. Sequences in which the WT splice sites were not detected by the programme were excluded from sensitivity and specificity calculation for the programme. In the initial calculation of sensitivity, variants that were predicted to cause a reduction in the splice site score, or completely abolished the site, were considered positive; however, in determining the optimal cut-off value in % score change, only variants with a % score change above a predefined threshold value were considered positive. When two programmes were used, variants were considered positive if one or more of the programmes predicted a reduction in the splice site score in excess of the cut-off value; when three programmes were used, variants were considered positive if two or more programmes predicted a reduction in the splice site score in excess of the cut-off values.

Receiver Operating Characteristic (ROC) curve analysis was performed using MedCalc for Windows, version 14.12.0 (MedCalc Software, Ostend, Belgium), which is available at http://www.medcalc.be/. MedCalc offers nonparametric ROC analysis with estimation of the Area Under the ROC Curve (AUC) based on the method developed by Hanley and McNeil [29]. Statistical significance was calculated using *t*-test by GraphPad Prism version 6 for Windows (GraphPad Software, San Diego, California, USA), available at http://www.graphpad.com/.

## 3. Results

**Figure 1:**
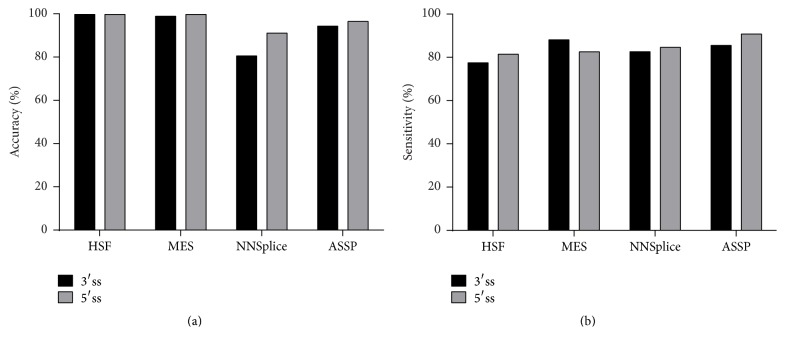
Overall performance of the splice site prediction programs. (a) Accuracy in predicting WT splice sites in reference sequences. (b) Sensitivity of detecting splice site change using 222 pathogenic mutations.

Prior to variant analysis, the programmes were compared in their ability to detect the wild type (WT) splice sites in reference sequences for all the genes of interest. As shown in Figure 1(a), NNSplice had the lowest accuracy in detecting the WT splice site for both 3′ splice sites (3′ss; −9 to −3′; splice acceptor sites) and 5′ splice sites (5′ss; +3 to +7; splice donor sites), with an accuracy of 80.76% and 91.4%, respectively. HSF had the highest accuracy in identifying the WT site, followed by MES and then ASSP.

Figure 1(b) summarizes the sensitivity of the programmes in predicting the impact on splicing of the 222 pathogenic variants used in this study. Sensitivity is measured as the ability of each program to predict a reduction in splice score by the pathogenic variant compared to that of WT. ASSP gave the highest sensitivity scores of 85.83% and 91.01% for 3′ss and 5′ss, respectively. MES gave the second highest sensitivity scores, with values of 88.37% and 82.8% for 3′ss and 5′ss, respectively. HSF have the lowest sensitivity among the four programmes tested.

**Figure 2:**
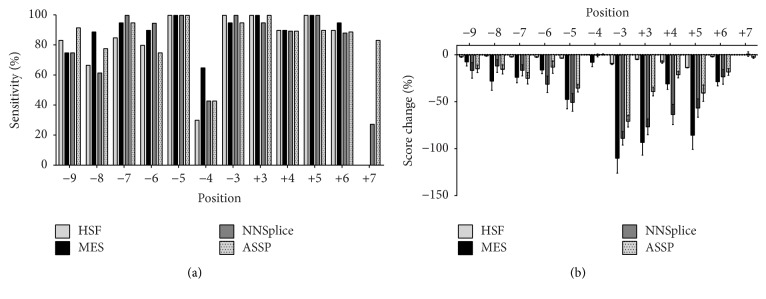
Splice site prediction relative to the position of mutations. (a) Sensitivity of splice site change detection relative to position; (b) % score change of the splice site caused by the mutations relative to position. In this calculation of sensitivity, mutations that were predicted to reduce splice site score compared to that of WT are considered positive.

Figure 2(a) shows a plot of the sensitivity of the programmes in predicting pathogenicity at defined positions within the 5′ss and 3′ss regions. All four programmes exhibited high (>90%) sensitivity at positions closest to splice junctions such as −3, −5, +3, +4, and +5, with the exception of position −4. For 3′ss, all programmes showed low sensitivity at the −4 position, with MES showing the highest sensitivity among the programmes at 65%. In the case of HSF, only 30% sensitivity was achieved. For positions further from the splice site (−6, −7, −8, and −9), MES and ASSP showed higher sensitivity than HSF and NNSplice, with the latter two programmes showing low sensitivity at −8.

For 5′ss, the highest sensitivity was achieved at +6 (>88% by all four programmes). The sensitivity was low at position +7; this conclusion is similar to the observation made by Hellen.

The mean % score change relative to the positions of mutations mirrored that of sensitivity, with all four programmes giving high % score changes at positions −3, −5, +3, +4, and +5 (Figure 2(b)). HSF gave a low dynamic range of % score change, with the mean % score change within 10% for all positions except −3 and +5. The other three programmes gave a greater range of score changes, with a general trend of a decrease in the mean % score change as the variant position moved upstream and downstream within the 3′ss and 5′ss, respectively. This general conclusion did not apply for position −4. All the programmes exhibited low score changes for positions −4 and +7. Overall, the data showed that the* in silico* analyses of variants at positions −4 and +7 were uninformative. For this reason, positions −4 and +7 were excluded from subsequent analyses in this study.

**Figure 3:**
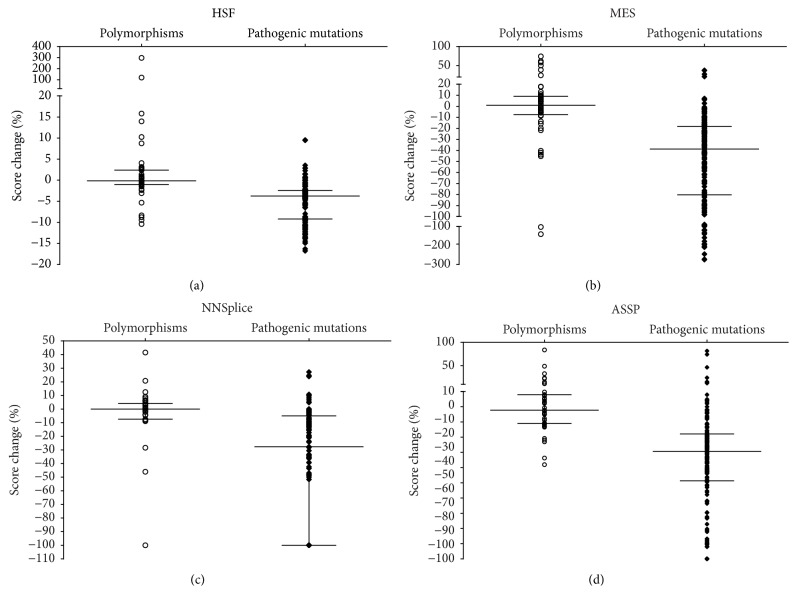
Dynamic range of the predicted splice site score change. The bars from top to bottom represent the 75% percentile, median, and the 25% percentile, respectively. The means of predicted score change for polymorphisms were significantly lower than disease-causing mutations by all four programmes (*P* < 0.05).

In this study, a collection of 50 benign polymorphisms that lay in the consensus splice site regions was obtained and subjected to analysis.  Figure 3 summarizes the % score change of 50 polymorphisms compared to those of the pathogenic variants. The predictions showed significantly reduced splice site score changes compared to those of pathogenic variants. Interestingly, HSF showed the lowest score reduction for both polymorphism and pathogenic variants compared to the remaining three programmes, with a median and 25% percentile % score change of −0.14% and −1.032 for the polymorphisms, respectively, and −3% and −9.2% for the pathogenic mutations, respectively.

**Figure 4:**
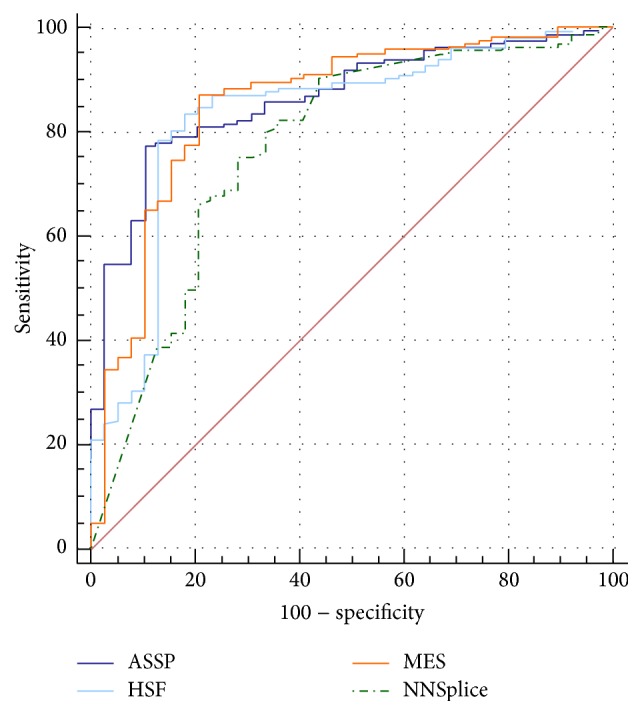
ROC curve comparison of the four prediction programmes. Each ROC curve shows a trade-off between sensitivity and specificity for each programme over a range of decision threshold values. The perfect ROC curve would align with the upper left corner, at a sensitivity of 100% and a false-positive rate of 0%. Therefore, the closer the ROC curve is to the upper left axis, the greater the accuracy of the prediction programme is.

Several studies have compared the performance of the splice prediction programmes using a defined decision threshold cut-off value [17, 19]. However, the optimal cut-off values may differ depending on the programme used; therefore we compared the performance of the prediction programmes independent of the cut-off threshold and plotted the data as ROC curves (Figure 4). The ROC curve plots the true positive rate against the false-positive rate (or sensitivity versus 1 − specificity) at various decision threshold settings and is commonly used as a tool to assess the performance of diagnostic tests. The accuracy of prediction programmes can be compared by calculating the AUC, which is a measure of the overall accuracy of a diagnostic test [30]. As shown in Table 1, MES and ASSP have the highest AUC, followed by HSF and then NNSplice. The ROC data suggest that MES and ASSP are the best of the four prediction programmes.

**Figure 5:**
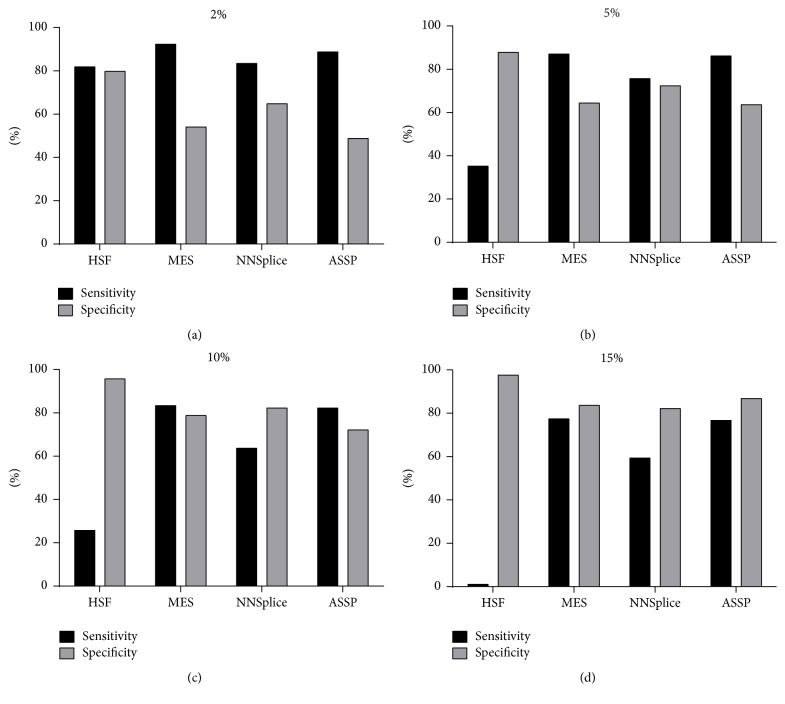
Sensitivity and specificity of data when analysed using a predefined threshold value for the % score change.

At present, there are no guidelines regarding the cut-off value for a splice site score change based on* in silico* predictions, although Houdayer et al. have suggested a cut-off of a 10% change using the MES programme. The use of 10% as cut-off value has also been suggested for HSF [24]. However, our dataset of % score change generated by HSF for pathogenic variants has a median value of less than 10%, suggesting that a lower cut-off value may be needed for this programme. We analysed our data using four different cut-off values of 2%, 5%, 10%, and 15% (Figure 5). For HSF, 2% was the optimal cut-off value, at which sensitivity and specificity of 82.11% and 81.25%, respectively, were achieved. The sensitivity of HSF decreases when using cut-off values greater than or equal to 5%, which can be attributed to the low score change generated by HSF. For MES and ASSP, 10% appears to be the optimal cut-off value. At 10%, MES achieved a sensitivity and specificity of 83.6% and 79.2%, respectively. ASSP achieved a sensitivity and specificity of 82.5% and 72.3%, respectively. NNSplice showed optimal sensitivity of 75.9% and specificity of 72.5% using a cut-off value of 5%. Although HSF achieved high sensitivity at a cut-off value of a 2% splice site score change, the low dynamic range of % score change it predicted may involve a challenging differentiation between pathogenic variants and polymorphisms.

**Figure 6:**
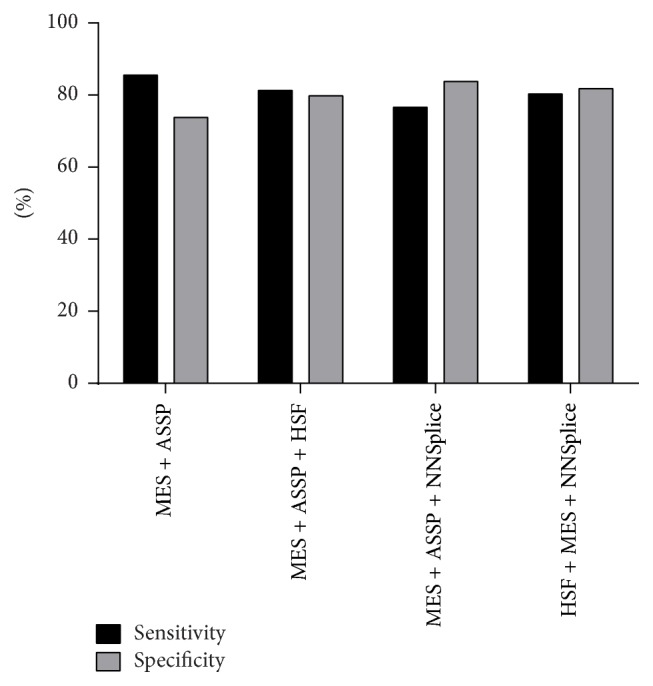
Performance of a combination of the splice site programs. Prediction programmes were used with the following cut-off threshold for % score change: HSF, 2%; MES, 10%; NNSplice, 5%; and ASSP, 10%. When two programmes were used, variants were considered positive if one or more of the programmes predicted a reduction in the splice site score in excess of the cut-off value; when three programmes were used, variants were considered positive if two or more programmes predicted a reduction in the splice site score in excess of the cut-off values.

The practice guidelines published by the UK Clinical Molecular Genetics Society and the Dutch Society of Clinical Genetics Laboratory Specialists suggest the use of at least three different programmes for the predictive analysis of splice variants [31]. In light of these guidelines, we assessed the sensitivity and specificity of different combinations of three programmes compared to using the best two (MES and ASSP) (Figure 6). Compared to the use of MES/ASSP only, all combinations of any three of the four programmes used here resulted in lower sensitivity but slightly improved specificity. The combination of three programmes with the highest sensitivity achieved was MES/ASSP/HSF.

## 4. Discussion

The expanding dataset of novel variants identified near splice site junctions demands consensus guidelines for* in silico* splice site prediction analysis. To date, there are few studies that have evaluated the currently available bioinformatic tools for splice site variant analysis. The study reported here evaluated four splice site prediction programmes (HSF, MES, NNSplice, and ASSP) in terms of their sensitivity and specificity regarding variants within splice site consensus regions. The data suggests that MES and ASSP are the two best performing programmes. Additionally, this study showed that the accuracy of splice site prediction is strongly affected by the level of conservation at a particular position.

Previously, Hellen suggested that the analysis of variants that lay outside positions −10 and +7 would be inaccurate in terms of predicting their effect on splicing. Additionally, Desmet et al. showed that the analysis of variants at positions +3 and +5 exhibited higher sensitivity compared to the rest of the consensus region (excluding the invariant dinucleotide positions). Our study examined individual positions within the splice site regions of −9 to −3 and +3 to +7 in terms of sensitivity in identifying variants as pathogenic. The highest sensitivity was obtained at positions adjacent to the splice junction such as +3, +4, +5, −3, and −5 but with a general decrease in splice site score change as variants lay further from splice junctions. Predictions for positions −4 and +7 are the least accurate for 3′ss and 5′ss, respectively. Variants located at position −4 resulted in poor predictions by all programmes, with the highest sensitivity being 65% by MES. The other three programmes gave sensitivities below 50% for the pathogenic mutations. The lack of sensitivity in identifying variants as pathogenic at position −4 could be attributed to the lack of sequence conservation at this position, which is located between the consensus triplet CAG preceding the intron:exon boundary and the pyrimidine tract further upstream. Although this position is thought to be involved in intron recognition, the analysis of a set of the U2-type introns from mammalian species shows that the four nucleotides are present at similar frequencies at this position [22]. Another position that showed low sensitivity was +7, with only NNSplice and ASSP being able to predict an effect on splicing. Similarly, a lack of conservation was observed at position +7 among U2-type intronic sequences [22]. These data suggest that, for poorly conserved positions such as −4 and +7, bioinformatic analysis may be uninformative and caution must be exercised in result interpretation in a diagnostic setting.

The ROC analysis suggests that MES and ASSP are the two best performing programmes. This is consistent with finding from Jian et al. [20], which showed that MES is the second best program amongst the eight programmes tested. The highest rated program from Jian et al. is PWM, which is integrated in the Alamut Suite. As it is not available publicly, a direct comparison to ASSP cannot be performed. Although the rating of performance is consistent with Jian et al., the AUC values for the same programmes are different, with Jian et al. reporting higher AUC values. This could be attributed to the difference in selection of variants. A close examination of data from Jian et al. revealed that the majority of the disease-causing mutations tested lay at the last base in the exonic region, followed by position +5. The majority of polymorphisms are located at position +3. This study has shown that position of variants have a significant impact on prediction accuracy, with positions +3 and +5 giving high sensitivity of prediction. The variants in this study are evenly distributed across the regions −9 to −3 and +3 to +7. Therefore, the difference in AUC values compared to Jian et al. is likely to be due to positioning of the variants rather than the scale of dataset. Jian et al. have developed two ensemble learning algorithms for splice predictions [20]. Although its data is integrated into the dbNSFP v3.0 database, the algorithms are not available publicly for further evaluation.

The interpretation of a splice site prediction relies on the comparison of splice site scores generated using the WT and the variant sequences. Currently there is no consensus on the cut-off values for each programme, although a score reduction of 10% seems to be the most commonly used [17, 19]. As each programme uses different models to generate a splice site score, we considered that the extent of a score change rather than an absolute number should best reflect the efficacy of the programme and that this change (as a % score change) needed to be determined for each programme. The results presented here suggest that a value of 10% may be a good cut-off value for MES and ASSP, but the optimal cut-off values for HSF and NNSplice are lower. Desmet et al. suggested 10% as the cut-off value for HSF; however, the majority of intronic mutations reported by Desmet et al. comprised variants located at invariant dinucleotide positions as well as those occurring at positions +3, +4, and +5, which are likely to result in a high % score change. The score changes for positions +3, +4, and +5 reported by Desmet et al. were 14%, 7%, and 14%, respectively. The mean % score change in our study is consistent with those reported by Desmet et al. for positions +4 and +5, but HSF predictions for variants in the remaining positions in the splice site consensus regions generated much lower % score changes and the use of 10% as cut-off values for those positions may not be optimal. In the case of MES, score changes of 10%, 15%, and 20% have been suggested [17, 18, 32]. We recommend the use of 10% as the cut-off value for MES in order to ensure higher sensitivity and so reduce the chance of false negative predictions.

MES has been recommended by previous studies [18, 19]. However, some studies accessed MES via the commercial Alamut interface, whereas this study accessed MES via the free HSF website. Data from Hellen showed that MES from the commercial package exhibited higher sensitivity than the free stand-alone source [19]. It remains unclear why MES from the Alamut interface gave higher sensitivity than the free stand-alone source as described by Hellen, and it remains uncertain whether results obtained using MES from the Alamut interface would differ from those obtained in this study.

The analysis of variants in this study achieved sensitivity and specificity values of 85.79% and 74%, respectively, using a combination of MES and ASSP with a cut-off % score change of 10%. The addition of a third programme slightly reduced the sensitivity of the* in silico* analysis but marginally improved the specificity. Hellen showed that using three programmes did not improve the accuracy of* in silico* predictions compared to using one best programme. Although the choice of adding a third programme is up to users, the addition of HSF to the MES/ASSP combination is simple as the HSF website performs analysis using HSF and MES simultaneously. In this study, NNSplice has the lowest prediction accuracy from the ROC curve analysis. Additionally, it has the lowest sensitivity to detect the WT splice site, rendering analysis of many sequences uninformative. Therefore, we recommend the addition of HSF instead of NNSplice as the third programme for* in silico* analysis and that caution must be exercised when relying on the use of NNSplice alone.

Several questions were not addressed in this study. Firstly, the variants analysed were not implicated in activating cryptic splice site. Desmet et al. have suggested that the best programme for cryptic splice site analysis may differ from that used to examine variants that affect WT sites only. The analysis of cryptic splice sites is more complex as several factors need to be considered such as the location of the potential new site, the distance to the exon boundary, and signal strength of the splice branch point. It has been suggested that currently available programmes cannot perform reliable analysis with such complex parameters [17]. Therefore, the predictive analysis of such variants requires further investigation.

Some polymorphisms were predicted to affect splicing, shown by high % score change. It has been suggested that the specificity of* in silico* prediction is affected by the WT splice signal, as reliability of prediction reduces when the WT consensus sites are poorly defined by the programme [18]. It is possible that the polymorphisms predicted to induce large score change in this study were in exons where the WT consensus sequence was weak or not well detected by the programme. This is supported by the observation that NNSplice predicted most large score reduction for polymorphisms (Figure 3), and it has been shown to have the lowest sensitivity in detecting WT splice sites in this study. Further investigation is needed using a larger dataset and comparison of splice signal of the WT sites in a gene-specific manner.

After applying the cut-off values determined for each program, there are three polymorphisms that showed % score change higher than the cut-off values by all four programmes (Supplementary Table  2). Without direct RNA study, it is uncertain whether they affected splicing; therefore the true accuracy of the splice prediction programme cannot be assessed. Their classification as benign polymorphisms may be due to lack of clinical evidence and presence in healthy individuals. For example, one of the three polymorphisms, rs41274636, is present in 1000 Genome phase 3 data at an allele frequency of 8.57% [33]. Given its high allele frequency it is unlikely to be pathogenic. It remains possible that a benign polymorphism may affect splicing in regions that are less important for protein function or the level of WT or other in-frame transcripts are sufficient to maintain the gene's function. For example, a recent study suggests that any* BRCA1* allele that permits 20–30% tumor suppressor activity may not confer* BRCA*-associated cancer risk [34]. The use of bioinformatic prediction is helpful to aid in clinical interpretation, but given the complex mechanisms of splicing in disease, multiple lines of evidence are needed. Experimental evidence on the variant's impact on splicing as well as the transcript's naturally occurring alternative splicing isoforms must be investigated for clinical evaluation. The limitations of present study reinforce the importance of obtaining RNA data in diagnostic laboratories.

Ideally, the evaluation of splice variants should be performed with a large set of newly identified variants from multiple genes along with experimental evidence of RNA splicing. With the increasing use of whole transcriptome shotgun sequencing (RNA-seq) and sharing of data between laboratories, such study can be performed in the near future.

## 5. Conclusions

This study evaluated the use of four currently available programmes to predict the effects of variants that lay within consensus splice site regions. Based on this evaluation, we recommend the use of MES/ASSP using a splice site cut-off value of 10%. In order to meet the best practice guidelines for splice site analysis, the use of three programmes is recommended; then the combination of HSF/MES/ASSP is optimal. Importantly, for poorly conserved positions (−4 and +7), the use of current bioinformatic tools should be considered uninformative.

## Supplementary Material

The Supplementary Table 1 lists the pathogenic mutations used for this study, whereas Table 2 lists the benign polymorphisms. All the sequence variants are provided with position, HGVS nomenclature and the gene involved. Prediction results from all four programmes using the optimal cut-off values determined from this study are provided.

## Figures and Tables

**Table 1:** Comparison of AUC data based on ROC curve analysis.

Programme	AUC	Standard error	95% confidence interval
HSF	0.834	0.0391	0.776 to 0.883
MES	0.878	0.0346	0.824 to 0.920
NNSplice	0.766	0.0462	0.702 to 0.823
ASSP	0.881	0.0268	0.828 to 0.922

## Abbreviations

HGMD: Human Gene Mutation Database
dbSNP: Database of Single Nucleotide Polymorphisms
HSF: Human Splice Finder
MES: Max Entropy Scan
ASSP: Alternative Splice Site Predictor
RNA: Ribonucleic acid
cDNA: Complementary DNA
mRNA: Mature ribonucleic acid
SSF: Splice Site Finder
PCR: Polymerase chain reaction
RT-PCR: Reverse transcription PCR
WT: Wild type
3′ss: 3 prime splice acceptor site
5′ss: 5 prime splice donor site
ROC: Receiver Operating Characteristic
AUC: Area Under the ROC Curve.
