# Supplementary material for: Evaluation of Bioinformatic Programmes for the Analysis of Variants within Splice Site Consensus Regions
Source: Adv Bioinformatics. 2016 May 24;2016:5614058. doi: 10.1155/2016/5614058 (PMC4894998; doi:10.1155/2016/5614058)
Supplement: Supplementary file 1 — The Supplementary Table 1 lists the pathogenic mutations used for this study, whereas Table 2 lists the benign polymorphisms. All the sequence variants are provided with position, HGVS nomenclature and the gene involved. Prediction results from all four programmes using the optimal cut-off values determined from this study are provided. [file 5614058.f1.pdf]

Supplementary Table 1. Pathogenic mutations

Position	Gene	Variant	HSF	MES	Nnsplice	ASSP
-9	CFTR	NM_000492.3: c.1585-9T>C	N	N	Y	N
-9	ETFDH	NM_004453.3: c.488-9T>C	N	N	N	Y
-9	F9	NM_000133.3: c.392-9T>G	Y	N	Y	Y
-9	HPRT1	NM_000194.2: c.533-9T>A	Y	Y	Y	Y
-9	HPRT1	NM_000194.2: c.533-9T>G	Y	Y	Y	Y
-9	MBTPS2	NM_015884.3: c.671-9T>G	Y	Y	N	Y
-9	MPZ	NM_000530.6: c.449-9C>T	N	N	N	N
-9	NXNL1	NM_138454.1: c.327-9G>A	N	Y	Y	N
-9	PARK2	NM_004562.2: c.535-9T>A	Y	N	Y	Y
-9	PMM2	NM_000303.2: c.640-9T>G	Y	Y	Y	Y
-9	RET	NM_020975.4: c.3188-9C>T	N	Y	N	N
-9	USH2A	NM_206933.2: c.2168-9T>G	Y	Y	Y	Y
-8	APC	NM_000038.5: c.1549-8A>C	N	N	N	N
-8	ABCB11	NM_003742.2: c.2012-8T>G	Y	Y	NA	NA
-8	ARSB	NM_000046.3: c.1143-8T>G	Y	Y	NA	Y
-8	ATP7B	NM_000053.3: c.2122-8T>G	Y	Y	Y	Y
-8	CFTR	NM_000492.3: c.274-8T>C	N	N	N	N
-8	CFTR	NM_000492.3: c.2658-8C>G	Y	Y	Y	Y
-8	DGUOK	NM_080916.2: c.592-8T>G	Y	Y	Y	Y
-8	DMD	NM_004006.2: c.1705-8T>C	N	Y	N	N
-8	F9	NM_000133.3: c.392-8T>G	Y	Y	Y	Y
-8	FBN1	NM_000138.4: c.3083-8T>G	Y	Y	Y	Y
-8	GATA6	NM_005257.5: c.1429-8T>G	Y	Y	NA	NA
-8	HBB	NM_000518.4: c.316-8T>G	Y	N	N	Y
-8	LDLR	NM_000527.4: c.1061-8T>C	N	N	N	N
-8	LDLR	NM_000527.4: c.1061-8T>G	Y	Y	N	Y
-8	MAP3K1	NM_005921.1: c.634-8T>A	Y	Y	NA	Y
-8	NEIL2	NM_145043.2: c.492-8C>T	N	N	N	N
-8	TCIRG1	NM_006019.3: c.2014-8T>C	N	N	Y	N
-8	TCIRG1	NM_006019.3: c.504-8G>A	N	N	NA	N
-7	ATP7B	NM_000053.3: c.2866-7T>C	N	N	N	N
-7	BRCA2	NM_000059.3: c.68-7T>A	Y	Y	Y	Y
-7	COL4A3	NM_000091.4: c.3518-7C>G	Y	Y	Y	Y
-7	CREBBP	NM_004380.2: c.4281-7C>G	Y	Y	Y	Y
-7	CYP21A2	NM_000500.7: c.293-7C>G	Y	Y	Y	Y
-7	DMD	NM_004006.2: c.2804-7G>A	N	Y	NA	N
-7	F7	NM_000131.4: c.431-7T>G	Y	Y	Y	Y
-7	GALT	NM_000155.3: c.821-7A>G	N	N	Y	N
-7	HBB	NM_000518.4: c.316-7C>A	Y	Y	N	Y
-7	HBB	NM_000518.4: c.316-7C>G	Y	Y	N	Y
-7	KCNH2	NM_000238.3: c.473-7C>A	Y	Y	N	Y
-7	LAMB3	NM_000228.2: c.299-7C>A	Y	Y	N	Y
-7	MEN1	NM_130799.2: c.655-7C>A	Y	Y	NA	Y
-7	MVK	NM_000431.3: c.769-7T>G	Y	N	N	Y
-7	MYH7	NM_000257.3: c.346-7T>C	N	N	N	N
-7	NF2	NM_000268.3: c.1575-7C>A	Y	Y	NA	Y
-7	PAH	NM_000277.1: c.1066-7C>A	Y	Y	NA	Y
-7	PKD2	NM_000297.3: c.2359-7T>A	Y	Y	Y	Y
-7	PTS	NM_000317.2: c.164-7T>A	Y	Y	N	Y
-7	RB1	NM_000321.2: c.2490-7T>G	Y	Y	Y	Y
-6	ABCA4	NM_000350.2: c.2919-6C>A	Y	Y	Y	Y
-6	ABCB11	NM_003742.2: c.3214-6C>G	Y	Y	Y	Y
-6	ABCC6	NM_001171.5: c.346-6G>A	N	N	Y	N

-6	AMN	NM_030943.3: c.1170-6C>T	N	N	N	N
-6	ATM	NM_000051.3: c.1066-6T>G	Y	Y	N	Y
-6	BRCA1	NM_007294.3: c.135-6T>G	Y	Y	Y	Y
-6	CAPN3	NM_000070.2: c.1355-6G>T	N	N	NA	N
-6	CDH1	NM_004360.3: c.2440-6C>G	Y	N	N	Y
-6	CFTR	NM_000492.3: c.2620-6T>C	N	N	N	N
-6	CFTR	NM_000492.3: c.274-6T>C	N	N	N	N
-6	CUL3	NM_003590.4: c.1207-6T>G	Y	Y	Y	Y
-6	DFNA5	NM_004403.2: c.991-6C>G	Y	Y	Y	Y
-6	DSPP	NM_014208.3: c.52-6T>G	Y	Y	Y	N
-6	FANCD2	NM_033084.3: c.1948-6C>A	Y	Y	Y	Y
-6	FAS	NM_000043.4: c.506-6C>G	Y	Y	Y	Y
-6	FERMT1	NM_017671.4: c.1140-6T>A	Y	Y	Y	Y
-6	FGFR1	NM_023110.2: c.2187-6C>T	N	N	N	N
-6	FKTN	NM_001079802.1: c.1045-6C>G	Y	Y	N	Y
-6	GCK	NM_000162.3: c.680-6C>A	Y	Y	Y	Y
-6	GHR	NM_000163.4: c.785-6T>A	Y	Y	Y	Y
-5	ABCC6	NM_001171.5: c.1868-5T>G	Y	Y	Y	Y
-5	ABCA4	NM_000350.2: c.769-5T>G	Y	Y	NA	Y
-5	ABCC8	NM_000352.4: c.1672-5C>G	Y	Y	Y	Y
-5	ADAR	NM_001111.4: c.2886-5T>C	N	Y	Y	N
-5	ATP7B	NM_000053.3: c.1708-5T>G	Y	Y	Y	Y
-5	ATRX	NM_000489.4: c.5273-5C>G	Y	Y	Y	Y
-5	BRCA1	NM_007294.3: c.4987-5T>A	Y	Y	Y	Y
-5	CLCN1	NM_000083.2: c.1167-5T>G	Y	Y	Y	Y
-5	COL1A1	NM_000088.3: c.1984-5C>A	Y	Y	Y	Y
-5	DKC1	NM_001363.4: c.85-5C>G	Y	Y	NA	Y
-5	DMD	NM_004006.2: c.1705-5T>G	Y	Y	Y	Y
-5	DPM1	NM_003859.1: c.373-5T>A	Y	Y	NA	Y
-5	DYNC2H1	NM_001080463.1: c.2346-5T>G	Y	Y	Y	Y
-5	FANCA	NM_000135.2: c.710-5T>C	Y	Y	Y	N
-5	FAS	NM_000043.4: c.569-5T>G	Y	Y	Y	Y
-5	FBN2	NM_001999.3: c.3974-5T>G	Y	Y	NA	NA
-5	FRMD7	NM_194277.2: c.206-5T>A	Y	Y	NA	Y
-5	GCH1	NM_000161.2: c.344-5T>G	Y	Y	NA	Y
-5	GNAL	NM_001142339.2: c.274-5T>C	N	Y	N	N
-4	ANTXR2	NM_058172.5: c.225-4G>A	N	N	N	N
-4	APOB	NM_000384.2: c.2068-4T>A	N	N	N	N
-4	ATP7A	NM_000052.6: c.2917-4A>G	N	N	Y	N
-4	CDKN2A	NM_000077.4: c.151-4G>C	N	N	N	N
-4	CFTR	NM_000492.3: c.2909-4A>G	N	N	Y	N
-4	CFTR	NM_000492.3: c.3140-4A>G	N	N	NA	NA
-4	DHCR7	NM_001360.2: c.99-4G>A	N	N	N	N
-4	EMX2	NM_004098.3: c.407-4G>T	N	Y	N	N
-4	F9	NM_000133.3: c.392-4T>C	N	N	N	N
-4	F9	NM_000133.3: c.839-4A>G	N	Y	Y	N
-4	GNE	NM_001128227.2: c.1505-4G>A	N	Y	NA	NA
-4	HNF4A	NM_175914.4: c.427-4G>A	N	N	NA	N
-4	IDUA	NM_000203.4: c.973-4G>A	N	N	Y	N
-4	LDLR	NM_000527.4: c.941-4G>A	N	N	NA	N
-4	MEFV	NM_000243.2: c.1760-4G>A	N	N	N	N
-4	NF1	NM_000267.3: c.2851-4T>G	N	N	N	N
-4	PPT1	NM_000310.3: c.363-4G>A	N	N	N	N
-4	PROC	NM_000312.3: c.71-4C>T	N	N	NA	N
-4	PROC	NM_000312.3: c.263-4G>A	N	N	N	N
-4	SLC12A3	NM_000339.2: c.1568-4G>A	N	N	NA	NA

-3	ABCB4	NM_000443.3: c.3280-3C>G	Y	Y	Y	Y
-3	ABCC6	NM_001171.5: c.3507-3C>A	Y	Y	N	Y
-3	ABCC6	NM_001171.5: c.3507-3C>T	Y	N	N	Y
-3	ABCC6	NM_001171.5: c.3634-3C>A	Y	Y	Y	Y
-3	ABCC8	NM_000352.4: c.3989-3C>G	Y	N	NA	Y
-3	ABCD1	NM_000033.3: c.901-3C>G	Y	Y	Y	Y
-3	ACVRL1	NM_000020.2: c.314-3C>G	Y	Y	Y	Y
-3	ACVRL1	NM_000020.2: c.626-3C>G	Y	Y	Y	Y
-3	AGK	NM_018238.3: c.424-3C>G	Y	Y	Y	Y
-3	ALG8	NM_024079.4: c.778-3C>A	Y	Y	Y	Y
-3	AMN	NM_030943.3: c.44-3C>G	Y	Y	Y	Y
-3	ANKS6	NM_173551.3: c.1973-3C>G	Y	Y	Y	Y
-3	APC	NM_000038.5: c.1409-3T>G	Y	Y	Y	Y
-3	APC	NM_000038.5: c.1744-3T>G	Y	Y	Y	Y
-3	APC	NM_000038.5: c.423-3T>A	Y	Y	NA	NA
-3	AR	NM_000044.3: c.1769-3C>G	Y	Y	Y	Y
-3	ARSA	NM_000487.5: c.1108-3C>G	Y	Y	Y	Y
-3	ATP7B	NM_000053.3: c.3061-3C>A	Y	Y	Y	Y
-3	ATP8B1	NM_005603.4: c.2932-3C>A	Y	Y	Y	Y
-3	BBS4	NM_033028.4: c.157-3C>G	Y	Y	Y	Y
3	ABCA4	NM_000350.2: c.66+3A>C	Y	Y	Y	Y
3	ABCA4	NM_000350.2: c.5312+3A>T	Y	Y	Y	Y
3	ABCB11	NM_003742.2: c.76+3G>T	Y	Y	Y	Y
3	ABCB11	NM_003742.2: c.150+3A>C	Y	Y	Y	Y
3	ACAD8	NM_014384.2: c.841+3G>C	Y	Y	Y	Y
3	ALDH5A1	NM_001080.3: c.1343+3A>T	Y	Y	Y	Y
3	AMH	NM_000479.3: c.412+3A>C	Y	Y	Y	Y
3	ATCAY	NM_033064.4: c.965+3G>T	Y	Y	Y	Y
3	BBS1	NM_024649.4: c.1110+3G>C	Y	Y	Y	Y
3	BLM	NM_000057.3: c.3210+3A>T	Y	Y	Y	Y
3	BLNK	NM_013314.3: c.47+3A>T	Y	Y	Y	Y
3	SCN1A	NM_001165963.1: c.2589+3A>T	Y	Y	N	Y
3	TPM1	NM_001018005.1: c.240+3A>G	Y	Y	N	N
3	BMPR2	NM_001204.6: c.418+3A>T	Y	Y	Y	Y
3	BRCA1	NM_007294.3: c.134+3A>C	Y	Y	Y	Y
3	NIPBL	NM_133433.3: c.3768+3A>T	Y	Y	Y	Y
3	NIPBL	NM_133433.3: c.5225+3A>C	N	Y	Y	Y
3	LAMA2	NM_000426.3: c.112+3A>G	N	Y	Y	Y
3	VHL	NM_000551.3: c.463+3A>G	Y	Y	Y	Y
3	VHL	NM_000551.3: c.463+3A>T	Y	Y	Y	Y
4	ABCB11	NM_003742.2: c.3213+4A>G	Y	Y	Y	Y
4	ABCC2	NM_000392.4: c.1031+4A>G	Y	Y	Y	Y
4	ALG8	NM_024079.4: c.673+4A>G	Y	Y	NA	NA
4	AMN	NM_030943.3: c.43+4A>G	Y	Y	Y	Y
4	APOB	NM_000384.2: c.904+4A>G	Y	Y	Y	Y
4	BBS1	NM_024649.4: c.1473+4A>G	Y	Y	Y	Y
4	BEST1	NM_004183.3: c.867+4G>A	N	N	NA	N
4	SLC12A3	NM_000339.2: c.1095+4A>G	Y	Y	Y	Y
4	G6PC	NM_000151.3: c.230+4A>G	Y	Y	Y	Y
4	GAA	NM_000152.3: c.2331+4A>G	Y	Y	Y	Y
4	RET	NM_020975.4: c.1879+4A>G	Y	Y	Y	Y
4	HADHB	NM_000183.2: c.209+4A>T	Y	Y	Y	Y
4	HNF1A	NM_000545.5: c.526+4A>G	Y	Y	Y	Y
4	HPRT1	NM_000194.2: c.609+4A>G	Y	Y	Y	Y
4	MAPT	NM_005910.5: c.915+4A>C	Y	Y	Y	Y
4	KIT	NM_000222.2: c.67+4G>A	N	N	N	N

4	PAX6	NM_000280.4: c.1183+4A>G	Y	Y	Y	Y
4	MLH1	NM_000249.3: c.790+4A>T	Y	Y	Y	Y
4	MAPT	NM_005910.5: c.915+4A>C	Y	Y	Y	Y
4	OTC	NM_000531.5: c.77+4A>C	Y	Y	N	Y
5	ABCC8	NM_000352.4: c.1923+5G>T	Y	Y	Y	Y
5	APC	NM_000038.5: c.531+5G>C	Y	Y	Y	Y
5	AR	NM_000044.3: c.1616+5G>C	Y	Y	Y	Y
5	AR	NM_000044.3: c.2449+5G>T	Y	Y	Y	Y
5	ANK1	NM_000037.3: c.27+5G>C	Y	Y	Y	Y
5	ASPM	NM_018136.4: c.2936+5G>T	Y	Y	NA	N
5	SCN1A	NM_001165963.1: c.3705+5G>C	Y	Y	Y	Y
5	VHL	NM_000551.3: c.340+5G>C	Y	N	Y	N
5	RS1	NM_000330.3: c.52+5G>C	Y	Y	Y	Y
5	SLC12A3	NM_000339.2: c.2368+5G>T	Y	Y	Y	Y
5	SLC2A2	NM_000340.1: c.775+5G>C	Y	Y	Y	Y
5	NF1	NM_000267.3: c.204+5G>C	Y	Y	Y	Y
5	NF1	NM_000267.3: c.586+5G>A	Y	Y	Y	Y
5	GCH1	NM_000161.2: c.343+5G>C	Y	Y	Y	Y
5	GCH1	NM_000161.2: c.509+5G>A	Y	Y	N	Y
5	GCH1	NM_000161.2: c.626+5G>A	Y	Y	Y	Y
5	RET	NM_020975.4: c.1879+5G>C	Y	Y	Y	Y
5	RET	NM_020975.4: c.2607+5G>A	Y	Y	Y	Y
5	RET	NM_020975.4: c.2939+5G>A	Y	Y	NA	Y
5	SDHD	NM_003002.3: c.169+5G>A	Y	Y	NA	Y
6	ABCA4	NM_000350.2: c.5584+6T>C	Y	Y	NA	Y
6	ABCC8	NM_000352.4: c.3329+6C>T	N	N	N	N
6	ACADM	NM_000016.5: c.708+6G>T	N	N	N	N
6	ADA	NM_000022.2: c.478+6T>A	Y	Y	Y	Y
6	ATP7A	NM_000052.6: c.1946+6T>C	Y	Y	Y	Y
6	ATP7A	NM_000052.6: c.2172+6T>G	Y	N	NA	NA
6	ATP7A	NM_000052.6: c.4226+6T>C	Y	Y	Y	Y
6	BMPR2	NM_001204.6: c.247+6T>G	Y	Y	NA	NA
6	BRCA1	NM_007294.3: c.4357+6T>C	Y	N	Y	N
6	BTK	NM_000061.2: c.974+6T>G	Y	Y	N	Y
6	CAPN3	NM_000070.2: c.1193+6T>A	Y	Y	N	Y
6	CFH	NM_000186.3: c.350+6T>G	Y	Y	Y	Y
6	COL3A1	NM_000090.3: c.582+6T>C	Y	Y	Y	Y
6	COL3A1	NM_000090.3: c.951+6T>C	Y	Y	Y	Y
6	PAH	NM_000277.1: c.1315+6T>A	Y	Y	Y	Y
6	PTCH1	NM_000264.3: c.1347+6G>A	N	Y	N	N
6	RB1	NM_000321.2: c.1695+6T>C	Y	Y	Y	Y
6	LAMA2	NM_000426.3: c.9211+6T>C	Y	Y	N	N
6	GH1	NM_000515.4: c.291+6T>C	Y	Y	Y	Y
6	GH1	NM_000515.4: c.291+6T>G	Y	Y	Y	Y
7	ABCC8	NM_000352.4: c.3867+7G>A	N	N	N	N
7	BRCA1	NM_007294.3: c.547+7G>A	N	N	N	N
7	CFTR	NM_000492.3: c.3367+7T>G	N	N	N	N
7	CHD7	NM_017780.3: c.6103+7A>G	N	N	N	N
7	FOXP3	NM_014009.3: c.816+7G>C	N	N	N	N
7	NCSTN	NM_015331.2: c.996+7G>A	N	N	N	N
7	NPHS1	NM_004646.3: c.1170+7C>T	N	N	N	N
7	PAH	NM_000277.1: c.1065+7C>A	N	N	N	N
7	PROS1	NM_000313.3: c.76+7A>G	N	N	N	N
7	PTEN	NM_000314.5: c.801+7A>G	N	N	Y	N
7	SCN1A	NM_001165963.1: c.2589+7A>G	N	N	N	N
7	VHL	NM_000551.3: c.340+7G>A	N	N	N	N

7	SDHB	NM_003000.2: c.200+7A>G	N	N	Y	N

N - predicted to not affect splicing using the cut-off value
Y - predicted to affect splicing using the cut-off value
NA - WT site not detected therefore analysis not available

Supplementary Table 2. Polymorphisms

Position	Gene	Variant	dbSNP	HSF	MES	Nnsplice	ASSP
-9	KRAS	NM_004985.4:c.451-9G>A	rs12313763	N	N	N	N
-9	MSH2	NM_000251.2:c.1511-9A>T	rs12998837	N	N	N	N
-9	CRBN	NM_016302.3:c.175-9T>C	rs1669321	N	N	Y	N
-9	FBN1	NM_000138.4:c.2855-9C>T	rs140590	N	N	N	N
-8	CREBBP	NM_004380.2:c.3837-8C>T	rs3025684	N	N	N	N
-8	RYR2	NM_001035.2:c.849-8T>C	rs16835237	N	N	N	N
-8	ACTN2	NM_001103.3:c.877-8C>G	rs2288601	Y	Y	N	N
-8	FBN1	NM_000138.4:c.3965-8T>C	rs140637	N	N	N	N
-8	RELN	NM_005045.3:c.4589-8T>G	rs362801	Y	N	Y	Y
-8	USH2A	NM_007123.5:c.3812-8T>G	rs646094	Y	N	Y	Y
-8	BRCA1	NM_007294.3:c.4485-8C>T	rs397507234	N	N	N	N
-8	RYR2	NM_001035.2:c.464-8A>C	rs10925391	N	N	N	N
-7	PCSK9	NM_174936.3:c.658-7C>T	rs2483205	N	N	N	N
-7	E1F2B4	NM_015636.3:c.76-7G>A	rs7602534	N	N	N	N
-7	MYO7A	NM_000260.3:c.5857-7A>T	rs1320703	N	N	N	N
-7	SEC23B	NM_006363.4:c.1405-7C>T	rs2273525	N	N	N	N
-6	RYR2	NM_001035.2:c.13783-6A>G	rs790901	N	N	N	N
-6	ALD	NM_000033.3:c.1489-6delC	rs398123101	Y	N	N	Y
-6	SCN5A	NM_000335.4:c.2788-6C>T	rs41260344	N	N	Y	N
-6	NKH	NM_000170.2:c.2316-6_2316-5insA	rs3215923	N	Y	N	N
-6	NDRG1	NM_001135242.1:c.64-6T>C	rs2272653	N	N	N	N
-5	MYH9	NM_002473.4:c.2038-5T>C	rs8137674	N	N	N	N
-5	ACTA1	NM_001100.3:c.130-5T>C	rs11803533	N	N	Y	N
-5	OTOA	NM_144672.3:c.1630-5C>T	rs215901	N	N	N	N
-5	MYO7A	NM_000260.3:c.593-5C>T	rs762666	N	N	N	N
-5	INVS	NM_014425.3:c.1235-5T>C	rs2245216	N	Y	Y	N
-5	MLYCD	NM_012213.2:c.642-5C>T	rs2278037	N	N	N	N
-5	EPCAM	NM_002354.2:c.492-5T>C	rs78608315	N	N	Y	Y
-5	NDRG1	NM_006096.3:c.756-5C>T	rs2227262	N	N	N	N
-3	ATP13A2	NM_022089.2:c.3084-3C>T	rs7531163	Y	N	N	Y
-3	ABCA4	NM_000350.2:c.6730-3T>C	rs1800717	N	N	N	N
-3	SCN2A	NM_021007.2:c.1035-3T>C	rs2121371	N	N	N	N
-3	LQT3	NM_000335.4:c.1141-3C>A	rs41312433	N	N	N	N
**3**	**ARFGEF2**	**NM_006420.2:c.423+3A>G**	**rs2295029**	**Y**	**Y**	**Y**	**Y**
3	TMPO	NM_001307975.1:c.959+3_959+4insT	rs34449077	N	N	N	N
**3**	**PCDH15**	**NM_033056.3:c.157+3A>G**	**rs41274636**	**Y**	**Y**	**Y**	**Y**
3	USH2A	NM_206933.2:c.15297+3A>G	rs57754754	N	Y	Y	N
4	OPA1	NM_130831.2:c.762+4T>C	rs166850	N	N	N	Y
4	CRYAB	NM_001289807.1:c.324+4T>G	rs11603779	N	N	N	N
4	HADHB	NM_000183.2:c.1149+4A>T	rs2303893	Y	Y	N	Y
**4**	**CBL**	**NM_005188.3:c.869+4A>G**	**rs77284821**	**Y**	**Y**	**Y**	**Y**
4	MYO6	NM_004999.3:c.1546+4_1546+5del	rs138200430	N	N	N	N
4	HFE	NM_001010980.4:c.-7-13380T>C	rs7515611	N	N	N	N
5	GALC	NM_000153.3:c.1834+5C>G	rs448805	N	N	N	N
5	NEBL	NM_006393.2:c.1008+5A>G	rs703089	N	N	N	N
5	GNPTAB	NM_024312.4:c.3135+5T>C	rs759935	N	N	N	Y
5	SYNE2	NM_182913.2:c.235+5C>T	rs1152590	N	N	N	Y
6	CHRNB1	NM_000747.2:c.610+6T>C	rs2302765	Y	Y	N	Y
6	FLCN	NM_144997.5:c.1062+6C>T	rs8065832	N	N	N	N
6	ATP7B	NM_000053.3:c.3903+6C>T	rs2282057	N	N	N	N

Bold: indicate polymorphisms which had %score change above cut-off for all four programmes.
N - predicted to not affect splicing using the cut-off value
Y - predicted to affect splicing using the cut-off value
NA - WT site not detected therefore analysis not available
